# Thermodynamic Driving Forces for the Self-Assembly of Diblock Polypeptoids

**DOI:** 10.1021/acsnano.3c12228

**Published:** 2024-05-29

**Authors:** Xubo Luo, Tianyi Yu, Nan K. Li, Ronald N. Zuckermann, Xi Jiang, Nitash P. Balsara, David Prendergast

**Affiliations:** † Materials Sciences Division, Lawrence Berkeley National Laboratory, Berkeley, California 94720, United States; ‡ The Molecular Foundry, Lawrence Berkeley National Laboratory, Berkeley, California 94720, United States

**Keywords:** polypeptoid, molecular dynamics simulation, self-assembly, nanofiber, nanosheet

## Abstract

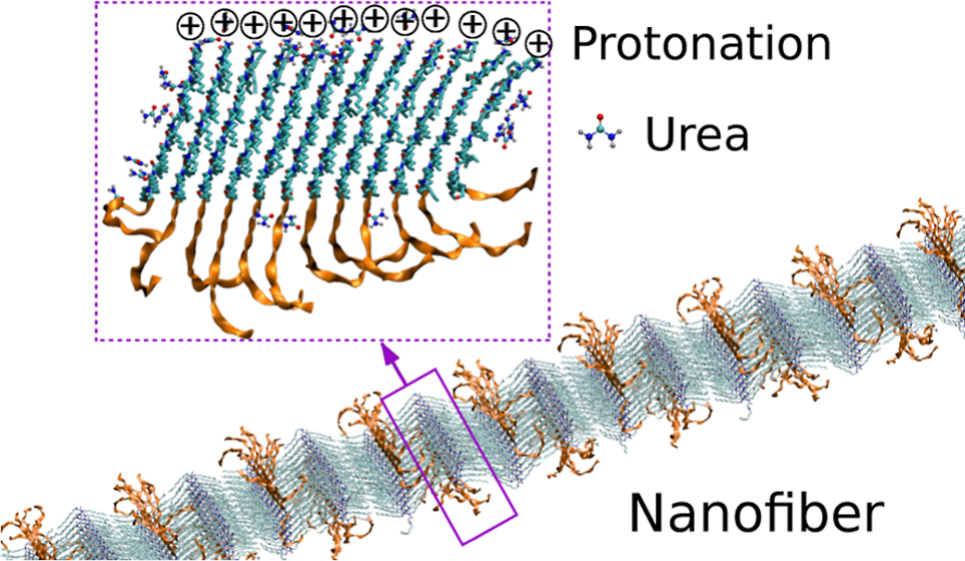

Peptoid polymers with sequence-defined side chains are observed to self-assemble into a variety of structures spanning nanometer and micron scales. We explored a diblock copolypeptoid, poly(*N*-decylglycine)_10_-*block*-poly(*N*-2-(2-(2-methoxyethoxy)ethoxy)-ethylglycine)_10_ (abbreviated as Ndc_10_-Nte_10_), which forms crystalline nanofibers and nanosheets as evidenced by recent cryo-transmission electron microscopy, atomic force microscopy, X-ray diffraction, and calorimetry. Using all-atom molecular dynamics simulations, we examined the thermodynamic forces driving such self-assembly and how nanoscale morphology is tailored through modification of the N-terminus or via the addition of small molecules (urea). We have found that the hydrophobic Ndc domain alignment is key to the formation of molecular stacks whose growth is limited by electrostatic repulsion between protonated N-termini. These stacks are the building blocks that assemble via cooperative van der Waals attraction between the tips of extended decyl side chains to form nanofibers or nanosheets with a well-converged intermolecular interaction energy. Assemblies are significantly more stable in urea solution due to its strong attraction to the peptoid–solvent interface. Isolated peptoids exhibit curved all-*cis* backbones, which straighten within molecular stacks to maximize contact and registry between neighboring molecules. We hypothesize that competition between this attractive interaction and a strain cost for straightening the backbone is what leads to finite stack widths that define crystalline nanofibers of protonated Ndc_10_-Nte_10_. Growth is proposed to proceed through backbone unfurling via *trans* defects, which is more prevalent in aqueous solution than in THF, indicating a possible pathway to self-assembly under experimentally defined synthesis conditions (viz., THF evaporation).

## Introduction

Polypeptoids are polymers of N-substituted glycine, analogous to polypeptides but with side chains shifted from the α carbon to the amide nitrogen.^1^ As biomimetic polymers, polypeptoids have been studied for a wide range of biomedical and biotechnological applications.^2−4^ In some cases, their functionality is highly dependent on their self-assembled structures.^5−9^ Unlike polypeptides, polypeptoids do not possess –NH hydrogen bond donors or chiral centers in their backbones. Consequently, the self-assembly of polypeptoids is predominantly governed by intermolecular interactions, which are tunable by the side-chain chemistry and monomer sequence.^10^ Owing to efficient solid-phase and solution-phase polymerization methods, the monomer sequence can be precisely controlled for polypeptoids,^11,12^ and versatile nanostructures have been observed for various chemical structures, such as nanotubes, nanosheets, and nanobrushes.^13−16^

To understand the fine structure of polypeptoid nanocrystals, researchers have used high-resolution cryo-transmission electron microscopy (cryo-TEM), as well as X-ray/neutron scattering and atomic force microscopy.^17,18^ With advanced data science techniques for image reconstruction,^19,20^ cryo-TEM images can be further refined to extract details of the crystal lattice down to 2–3 Å resolution. In the past, the reconstructed two-dimensional (2D) cryo-TEM images have resolved side chains and backbones in projection for polypeptoid nanosheets.^13,21−23^ Most recently, 3-dimensional (3D) image reconstruction was successfully performed for polypeptoid nanofibers, which provided more details of the lattice interior and unexpected information about chain orientations.^24^ Despite the power of cryo-TEM image reconstruction, it is still dose- and resolution-limited for the acquisition of electron density maps of crystalline phases, and the acquisition of distinct atomic positions has relied, to date, on correlation with molecular dynamics (MD) simulations in each case. These experiment-inspired simulations permitted the assignment of chemically meaningful atomistic models consistent with the images and played an essential role in understanding the molecular basis for peptoid lattices.

To approach predictive modeling, all-atom and coarse-grained force fields have been parametrized for low-molecular-weight or homopolymeric peptoids to distinguish them from previously described peptides.^25−31^ The available parameters allow us to model systems of higher molecular weight and various side chains. Due to the extremely long time scale of the entire self-assembly process, MD simulations of peptoid assemblies to date have been initialized with preassembled nanostructures inspired by experiment (either X-ray scattering or cryo-TEM data), and consequently, such simulations can reach equilibrium within an acceptable computational time. Mannige et al. performed MD simulations for preassembled bilayer nanosheets of an amphiphilic ionic polypeptoid.^32^ Subsequent MD studies revealed that assembled polypeptoid backbones have linear and untwisted conformations (all *cis*-conformations) in nanosheets.^33^ This conclusion was widely accepted in other studies, where the peptoid lattices shared the same cis-sigma strand that packed face-to-face in rows.^13,21−24^

MD simulations can begin to probe the mechanistic pathway of self-assembly by modeling the final nanocrystal and its postulated intermediates. In a recent study, Zhao et al. applied this methodology to nanosheets comprising diblock copolypeptoids of six *N*-((4-bromophenyl)ethyl)glycine conjugated to a polar NH_3_(CH_2_)_5_CO tail.^34^ The researchers proposed a hierarchical pathway involving 1-dimensional (1D) rods as the step toward nanosheets. Accordingly, they investigated the preassembled nanorods and nanosheets for structural details and attempted to evaluate the stability/formation in different solvents by simulating the peeling off of a peripheral molecule or allowing an additional block to approach. This research studied the assembly mechanism despite some yet unresolved aspects, such as an overall evaluation of the conformations of all simulated structures and tracking of growth with respect to size, which remain to be developed.

**Figure 1:**
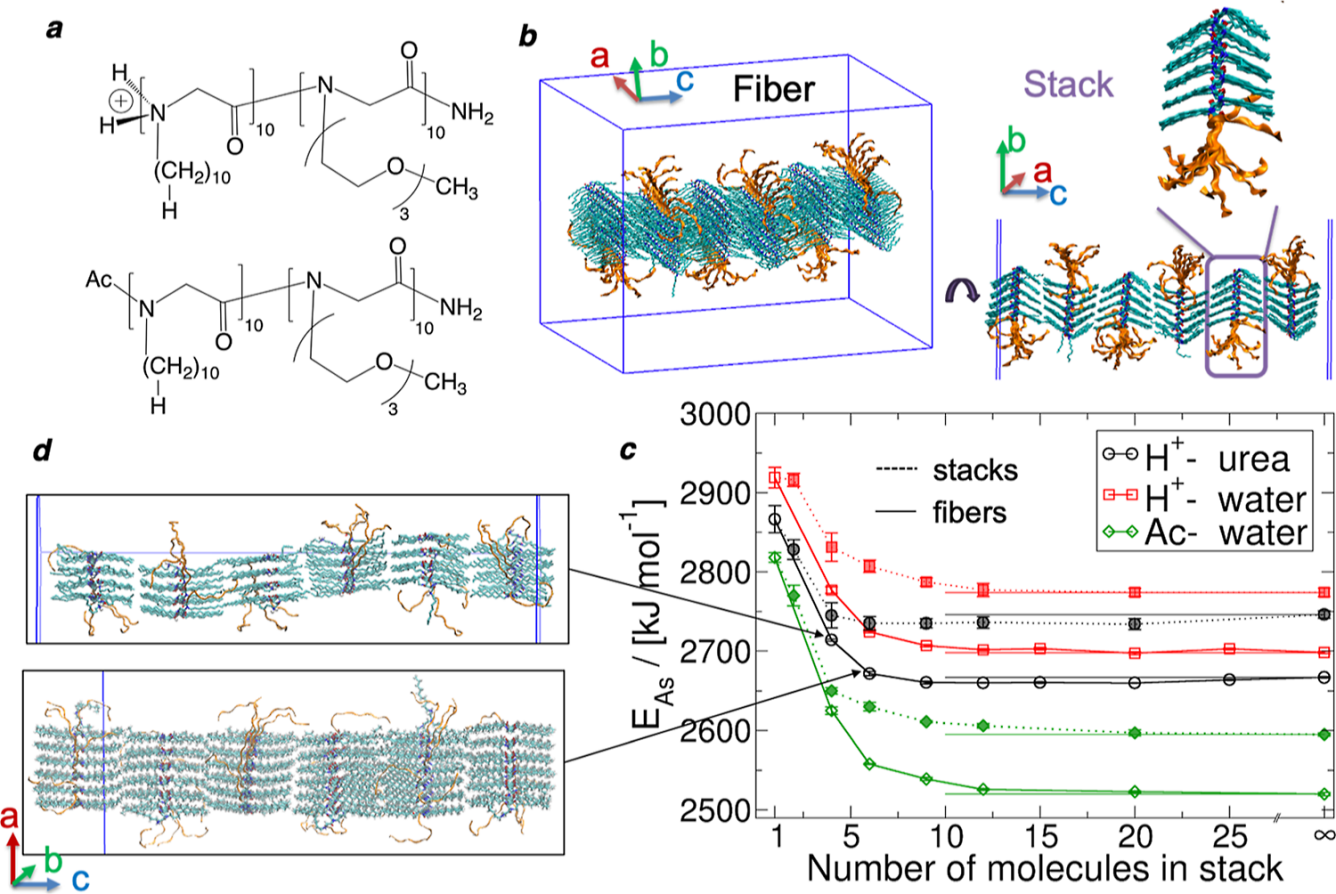
Structure and energetics: (a) molecular structure of the acetylated (Ac) and protonated (H^+^) Ndc_10_-Nte_10_ polypeptoids; (b) effective unit cell parameter labels (blue line: boundary of simulation box), indicating molecular stacking in the *a* direction, extended backbones in the *b* direction, and extended Ndc side chains in the *c* direction; (c) assembly energy per molecule as a function of the number of molecules stacked together (dashed line with filled symbols) vs extended fibers formed from the same (solid line with open symbols); (d) MD snapshots of fibers of various uniform molecular stacks (4, 6) assembled via contact between extended Ndc side chains.

This work performs molecular dynamics simulations for the amphiphilic diblock polypeptoid, poly(*N*-decylglycine)-*block*-poly(*N*-2-(2-(2-methoxyethoxy)ethoxy)ethylglycine), abbreviated as Ndc_10_-Nte_10_ (Figure 1a). This molecule has been reported to form crystalline nanofibers with interior structures of varying order or highly ordered crystalline nanosheets, depending on the solvent composition and peptoid N-terminus chemistry.^13,24^ These observations suggest that small changes in chemistry yield huge changes in morphology, yet the molecular basis is not understood. To mimic the hypothetical stages of nanocrystal growth, this work presents the results of all-atom molecular dynamics for fully solvated single molecules; preassembled nanofibers with varying molecular widths; nanosheets of infinite width; and molecular stacks, which are the primary building blocks of nanofibers. These structures are evaluated using their assembly energy per molecule to assess their relative stability. The simulations are performed in both urea solution and pure water for protonated N-termini and in pure water for acetylated N-termini. The different states probed in this study are expected to improve our understanding of the structural details, discover the molecular origins and driving forces for self-assemblies, unveil the impact of small motifs, and propose a hypothetical mechanism for self-assembly. The results may inspire rules for designing polypeptoids to achieve desired morphologies.

## Results

### Structure and Energetics of Polypeptoid Assemblies

A lot is known already about the nanocrystalline domains of assemblies of these crystalline peptoid nanostructures.^2^ The *a* lattice constant indicates the direction of molecular stacking where molecular backbones are aligned and in close proximity to one another; the *b* lattice constant is directed along the molecular backbone; and the *c* lattice constant defines the effective width of each molecular stack (or some small number of neighboring stacks). Molecular stacks have a finite number of molecules arranged along the *a* axis (Figure 1b, left). Nanofibers comprise many stacks, extended along the *c* axis, with neighboring stacks of molecules contacting at the tips of their extended side chains (Figure 1b, right). Stack neighbors may be aligned with their *b* axes to have the Nte-blocks alternating up, then down. Nanosheets are assemblies extended significantly along both the *a* and *c* axes. Our simulations start by constructing these initial structures (Figure S1), which are then equilibrated in solution to sample a thermodynamic ensemble at room temperature. We recognize that the imaging experiments are conducted under cryogenic conditions, but we make the approximation that freezing occurs swiftly enough to preserve room-temperature conformations. The relaxed structures (Figure 1b) are consistent with our prior observations of crystalline Ndc domains, showing the competence of our models.^24^

Figure 1c compares the assembly energy per molecule for acetylated and protonated Ndc_10_-Nte_10_ in water and in concentrated urea solution for varying numbers of molecules per stack. This is an effective solvation energy per molecule, defined for fair comparison between assemblies of different sizes fully solvated by different numbers of solvent molecules
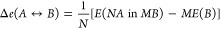
where *A* denotes a polypeptoid molecule, *B* denotes a solvent molecule, *N* is the number of *A*, and *M* is the number of *B* in a given simulation cell. *E*(*NA* in *MB*) is the potential energy of *N* polypeptoid molecules in *M* solvent molecules. A lower assembly energy per molecule indicates that the solvated nanostructure is more stable.

From Figure 1c, we note that there are significant energy gains associated with forming molecular stacks from isolated molecules. Approximately constant gains that promote fiber or sheet formation from corresponding stacks are associated with the fixed area of interaction per molecule between neighboring stacks. These energy gains for molecule-to-stack and stack-to-fiber are consistent with the differential scanning calorimetry,^24^ indicating this possible hierarchical mechanism in terms of thermodynamics. The depth of energy minima must be significant, given that these assemblies remain intact at finite temperatures; however, we do not see ordered assembly occur spontaneously in simulation (at least on typical computational time scales). Stable fibers form ordered stacks of at least 4 molecules despite their wavy shape, and the fibers become more ordered when there are 6 or more molecules stacked in the *a* direction (Figure 1d). Slight energy gains are observed for the formation of increasingly thicker stacks and, ultimately, sheets for Ac-terminated diblocks. On the contrary, protonated Ndc_10_-Nte_10_ in urea solution exhibits a local minimum in assembly energy per molecule at around 9–20 molecules, indicating that a nanofiber morphology (finite stacking along *a*) is thermodynamically preferred over the nanosheet morphology (extended stacking along *a*). We also notice the significant stabilization of protonated assemblies in concentrated urea solutions vs pure water. Although both solution conditions display similar energy gains when forming stacks and nanofibers from the protonated polypeptoid, a significant exception is the relative instability of the dimer stack in pure water, which is more stable in urea for protonated N-termini. This is also true for the acetylated dimers in pure water, which are more stable than single solvated molecules. The main features of the assembly energy profile are summarized in Table 1 for a clear demonstration of the impact of chemistry.

**Table 1:** General Features of Ndc_10_-Nte_10_ Assembly Energy Profile

system	overall stability	local minimum	energy gain for dimer
H^+^-urea	second	nanofibers of 9–20	yes
H^+^-water	third	not obvious	not significant
Ac-water	lowest	nanosheet	yes

### Molecular Structure of Polypeptoid Assemblies

**Figure 2:**
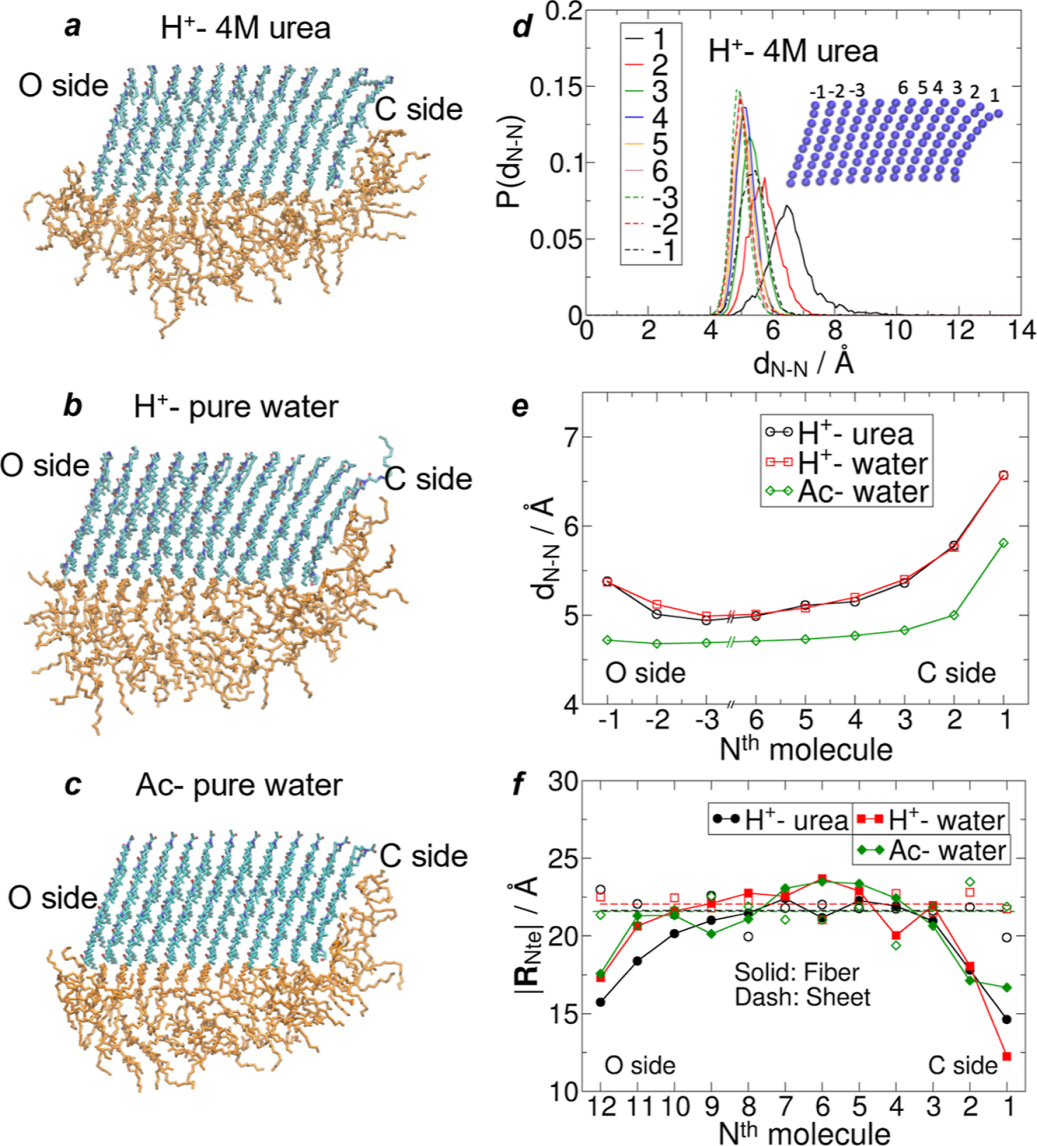
Assembly morphology. (a–c) Comparison of the assembled morphology of sliced stacks from the nanofibers of 12 Ndc_10_-Nte_10_ molecules in width. Viewed along the *c*-axis (extended side chains) to highlight backbone alignment/curvature in the Ndc block (blue) vs the disorder of the Nte block (orange). Backbone alignment is chemically organized with more hydrophilic carbonyl groups facing left (O-side) and more hydrophobic methyl groups facing right (C-side), with some protection from Nte; (d) distribution of N-termini separation numbered relative to the C-side; (e) comparison of the mode of N-termini separation between acetylated (Ac) and protonated (H^+^) polypeptoids; (f) average end-to-end length of the Nte block based on its position relative to the C-side, indicating stronger curvature at both ends, more significant at the C-side.

While the interior of molecular stacks exhibits nanocrystalline domains for their Ndc blocks, this crystallinity breaks down at the faces of these stacks in the *a* direction. A clear chemical distinction between each of these molecular stack faces results from the parallel orientation of the molecular backbones that present a carbonyl-rich O-side vs a methylene-rich C-side, which are, respectively, more hydrophilic and hydrophobic (as described previously in ref (24)). The simulated morphology shows that the C-side of each stack is partially protected by the Nte block of the outermost molecule (Figure 2a–c). This compact conformation likely presents a significant obstacle to the addition of molecules to the C-side and stack growth is much more likely at the more accessible O-side.

Protonated N-termini in self-assembled stacks are separated by ∼5 Å from their neighbors, and this introduces significant electrostatic repulsion between these groups. As noted in Figure 1c, in pure water, self-assembled protonated structures are significantly destabilized. On average, the separation of N-termini is larger when protonated (Figure 2d), which works against the close packing of neighboring backbones and leads to increased curvature of the backbone within the Ndc domain. Where these electrostatic repulsions are not balanced by neighboring peptoid molecules, *viz*. at the ends/faces of the stack, we observe much larger N-termini separations from their neighbors. The corresponding morphology is reminiscent of peeling layers at the stack surface, showing a gradual loss of order from the crystalline interior to the stack faces, similar to the outer pages of a well-worn, dog-eared book. This effect is mitigated for the acetylated N-terminus due to the absence of net charge, but some peeling is still observed at the C-side (Figure 2e), probably because of the tendency of the backbone to curve (see below) and the interaction with Nte. The latter is due to the unbalanced interactions of neighboring Nte and the large open face of Ndc, especially at the C-side, which is attractive for the Nte to fold over and touch that face (Figure 2f). In general, while the Nte block is always disordered, it is more extended in the interior of molecular stacks than in peripheral regions.

With the introduction of urea, the Ndc morphology remains effectively the same as in the pure water protonated case. However, the effective concentration of urea at the Nte and Ndc surfaces is about three to four times that in the bulk solution (Figure S2L), indicating the attraction of urea molecules to peptoid surfaces. In addition, urea induces a larger solvent-accessible surface area (SASA) in the Nte block (Figure S2R), which means that the Nte block is more solvated and probably explains the more flexibly curved Nte backbone on the O-side (Figure 2a). Despite the more flexible Nte, the SASA of the Ndc block alone is not much affected by urea. This means that the Nte coverage over Ndc only involves touching at points, which does not significantly exclude water molecules. However, the coverage by urea molecules in fact reduces the accessible surface area of Ndc and Nte blocks, as shown by the SASA when excluding the urea-occupied area, i.e., SASA for water only. Hence, urea passivates the hydrophobic surfaces, and this reduction in the water-accessible surface area results in an almost constant overall stabilization of assemblies (per molecule). Since the C-side and O-side faces define additional solvent-accessible surfaces for nanofibers, protection by urea is more beneficial for fibers of finite width than for infinitely wide nanosheets, which likely contributes to the local minima at 9–20 molecules per stack in urea solution, as shown in Figure 1c.

### Molecular Origins of Assembly Thermodynamics

**Figure 3:**
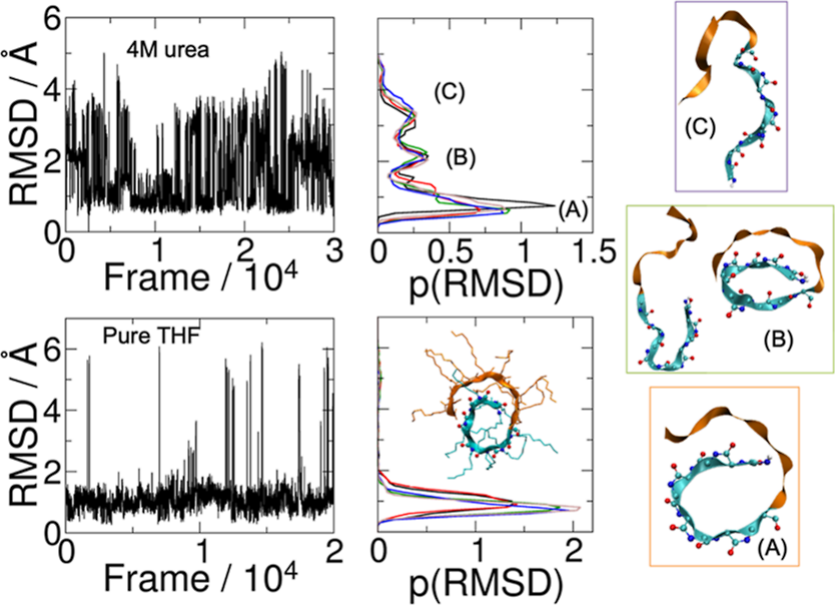
Conformational sampling of an isolated Ndc_10_-Nte_10_ molecule with protonated N-terminus in urea solution (top) and in pure THF (bottom). Left: root-mean-square deviations (RMSD) with respect to a coil conformation as a function of time during REST2 simulations. Middle: sampled probability distributions from these trajectories with primary peaks labeled (the single peak and its representative coiled conformation are indicated for THF). Right: representative conformations from urea solutions for the dominant coiled Ndc (A) and minority more open conformations with some *trans* defects (B,C) in urea solution.

Although straight backbones are observed in images of crystalline domains, we have noticed in our relaxed assemblies that a slight curvature of the backbone is evident, especially with protonated N-termini and always toward the C-side of a given stack. Since the assembly energetics are determined by reference to fully solvated single peptoid molecules, we effectively sampled the morphology of these species using molecular dynamics simulations coupled with the replica exchange with solute tempering 2 (REST2) technique.^35^ Single molecules exhibit two primary motifs: a coiled structure with the more hydrophobic Ndc block surrounded by Nte and more open structures with extended backbones, as shown in Figure 3 (We also observe a group of semicoiled structures in these statistics–more results are provided in Figures S3 and S4). Analysis of the distribution of root-mean-squared deviations (RMSD) from the coiled structure exhibits separated peaks, indicating that these two families define distinct local free-energy minima. It is notable that the aforementioned sigma-strand all-*cis* peptoid backbone conformation that leads to straight backbones in nanocrystals is approximately preserved in the coiled cases, while the extended and open structures have *trans* defects in their backbones. It is noticeable in Figure 3 that the open structure population is negligible when the protonated molecule is solvated in pure tetrahydrofuran (THF), which is the initial solvent in the experimental assembly process. Since open structures are likely required to initiate self-assembly (more details below), we conclude that this is favored as THF evaporates from mixed solutions of THF and water (as observed in experiment).

**Figure 4:**
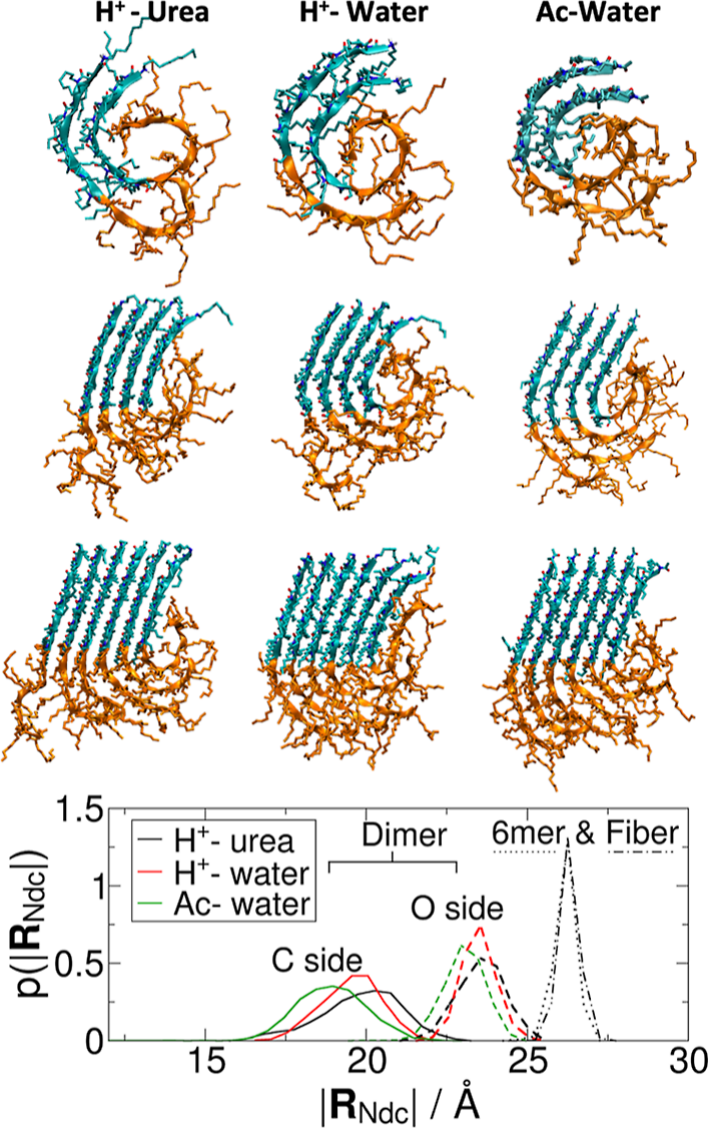
Evolution of backbone curvature with increasing stack thickness for protonated Ndc_10_-Nte_10_ in urea solution (left), in pure water (middle), and the acetylated case in water (right) for stacks of 2, 4, and 6 molecules (top to bottom). Bottom: distribution of the end-to-end distance of the Ndc for C-side and O-side in the dimer compared with the extended molecule in the middle of the 6mer stack and the fiber containing 12 molecules per stack in urea solution.

Examination of isolated molecules and molecular stacks indicates a preference for a curved *cis* backbone morphology. The smallest self-assembly, stable dimers, adopt the familiar oriented backbones, which have the O-side facing outward and the C-side inward with respect to their common curvature. An anticoiled molecule is observed on the C-side of the dimer, with its Nte block surrounded by its Ndc block, as shown in Figure 4. This is opposite to the expected coiled conformation observed for single molecules, which displays the opposite arrangement (Ndc surrounded by Nte). The formation of this anticoiled motif is common to the C-side of all molecular stacks and therefore dimer formation may present a bottleneck to self-assembly as anticoiled conformations are unstable and never observed to spontaneously form in solution. Interestingly, of all cases studied in this work, only the protonated peptoids in pure water exhibit no significant gain in energy upon dimer formation, which may explain why they do not exhibit an ordered interior structure in the nanofibers observed experimentally.^24^ Unlike dimer stacks, however, thicker molecular stacks exhibit successively straighter backbones, albeit with persistent curvature at the C (methylene) side of each stack. Figure 4 shows that the Ndc backbone in the middle of a stack becomes as straight as in extended nanofibers when the stack reaches six molecules. Recalling Figure 1d, stable, straight (nonwavy) fibers are only evident with six molecules per stack or more, and this coexistence of straight Ndc backbones and straight fibers indicates the necessity of backbone straightening for the connection of neighboring stacks.

Therefore, we conclude that the stack self-assembly is driven predominantly by attractive electrostatic and van der Waals forces that maximally align and overlap neighboring backbones and Ndc side chains, as observed experimentally. Unabated, these attractive forces should lead to extended nanosheets, as observed for acetylated peptoids. However, this attraction competes against the strain of straightening the naturally curved backbone conformation of each molecule, which can result in a local minimum in assembly energy for widths of 9–20 molecules when the attractive force is reduced by repulsive electrostatic interactions between protonated N-termini.

### Polypeptoid Growth Hypothesis

As discussed above, the assembly energy profile suggests a hierarchical assembly, with molecular stacks as the building blocks (growth along the *a* axis), comprising straightened peptoid backbones (defining the *b* axis), which then assemble into nanofibers/nanosheets (along the *c* axis) with a nearly constant energy gain. Despite challenges in tracking spontaneous assembly within finite simulation time scales, we can posit a hypothetical mechanism consistent with Sun et al. that molecular stacks may emerge from regions of concentrated polypeptoids, which could subsequently connect into nanofibers by contact between side-chain tips.^15^ The coiled all-*cis* conformation of isolated molecules may prevent the formation of ordered stacks, and the growth of molecular stacks most likely proceeds through the more open single-molecule population in aqueous solution. In an aqueous environment, the coexisting open and coiled peptoid molecules tend to agglomerate into peptoid-rich clusters, and the open molecules with some *trans* defects may easily align their respective backbones for the stable all-*cis* conformation dimer if permitted by the chemistry (nonprotonated N-terminus or stabilization in urea solution). The stack is more likely to grow on its O-side face due to its greater accessibility to other molecules. By contrast, the C-side is generally more curved, and its outermost Ndc block is often partially protected/blocked by its own Nte block when the surface is exposed to water molecules. This prevents the O-side of other molecules/stacks from joining in registry to the C-side face. As more peptoid molecules are added to stacks, the Ndc backbones become straighter, driven by the dominance of attractive intermolecular interactions over the tendency for intrinsic backbone curvature, and these more ordered stacks are able to connect into stable, straight nanofibers or nanosheets. Adjustment of this dominance of intermolecular attraction over intrinsic curvature is achievable by weakening the attractive component, achieved here through the introduction of repulsive electrostatic attraction, leading to finite width nanofibers, rather than extended nanosheets.

## Conclusions

This work utilized MD simulations to examine the relative stability of preassembled nanofibers, nanosheets, molecular stacks, and isolated molecules for protonated and acetylated Ndc_10_-Nte_10_ diblock polypeptoids. These simulations highlight the importance of molecular-scale details (edge effects and the peptoid–solvent interface) in revealing the thermodynamic and molecular origins of different assembled nanomorphologies in the Ndc_10_-Nte_10_ peptoid family, which are otherwise invisible to imaging experiments or scattering analysis. The final assembly is dictated by the presence or not of a local minimum in the profile of assembly energy. The subtle balance between attractive and repulsive forces in these assemblies indicates yet another means of controlling the final morphology–limiting growth in the *a* direction–either through chemical modification of the peptoid itself or its surroundings and highlights opportunities for dictating desirable nanomorphologies in this material class. We surmise that polypeptoid molecules first assemble into molecular stacks (along the *a* axis) with aligned backbones and Ndc decyl side chains, and these stacks connect at the tips of their extended side chains to form nanofibers (extended along the *c* axis). Growth along the fiber axis appears to be unlimited in our models, with a near-constant gain in assembly energy per molecule due to a reduction in the exposed hydrophobic surface area. The formation of each stack requires the existence of open (as opposed to coiled) backbone conformations for isolated molecules, which are observed to occur spontaneously in aqueous solution through a conformational switch to introduce some *trans* defects. The growth of ordered stacks most likely happens by molecular addition to their O-side faces in peptoid-rich regions/agglomerates, which should be facilitated by backbone opening/uncoiling and reorientation of peptoid molecules. Dimer formation could present a bottleneck to stack growth, as observed for protonated N-termini without stabilization from urea in solution. Future work will explore the emergence of ordered molecular conformations from agglomerates in solution, taking inspiration from the stable structures observed here.

## Methodology

### Classical MD Simulation

For polypeptoid molecules, a protonated or acetylated N-terminus was modeled to match previous experiments, with trifluoroacetate as the counterion for the former.^13,24,36^ The force field was the same as in our prior studies.^23,24^ The interactions of peptoid backbone atoms were modeled with the CGenFF-based force field by Weiser and Santiso,^28^ and the side chains and the counterions were modeled with the standard CGenFF.^37^ For the missing bonded and van der Waals parameters of the N-terminus and the counterion, an automatic tool was used to match the atom types and obtain the parameter set.^38,39^ The partial charges for these species were obtained using the RESP algorithm from the results of density functional theory calculations using TeraChem 1.93 at the B3LYP/6-311G** level.^40^ The obtained partial charges are listed in Figure S5 and Table S1. It is acknowledged that there are different ways to determine the partial charges when using CGenFF-based force fields.^27,37,41,42^ In this instance, our results and conclusions are not noticeably modified when compared to other methods for determining the partial charges for these ionic moieties, as shown in Figure S6 and Table S2 in the Supporting Information. The TIP3P model was adopted for water molecules. All MD simulations were performed using GROMACS (version 2019.2 or above).^43^ The cutoff distances for van der Waals and electrostatic interactions were set to 12 Å. Particle-mesh Ewald summation was selected for the long-range Coulombic interactions. The time step was set to 2 fs, and the bonds involving hydrogen atoms had fixed lengths using the LINCS algorithm.^44^ Classical MD simulations were performed for the nanocrystals with preassembled initial structures solvated in large supercells. We did not observe spontaneous self-assembly of peptoids during the typical submicrosecond lengths of trajectories for all-atom simulations.^31^ The preassembled structures were inspired by the reconstructed cryo-TEM images in our prior study, which showed that the Ndc blocks defined the crystal phase while the Nte blocks were amorphous with some solvent penetration.^24,36^ In our models, the Ndc chains were stacked with aligned backbones, which were initialized in the all-*cis* conformation, and the molecules were packed into stacks, as shown in Figure S1. The neighboring stacks were arranged into long fibers with the tips of Ndc-block decyl side chains in contact. This arrangement was carefully chosen so that all molecules were facing the same side in each stack, and the stacks were aligned for the best contact. To probe nanofiber stability with respect to width, we varied the number of molecules in each stack from 4 to 25. A model nanosheet of 12 molecules per stack was also prepared with the two side surfaces contacting each other at the periodic boundary. Based on our preliminary tests, a tilt angle of the peptoid backbone (*b* axis) of ∼65° resulted from relaxation of the nanosheet. All simulations started with 10 ns of Langevin dynamics in vacuum to achieve amorphous Nte blocks, while the Ndc blocks were fixed by a harmonic potential with a force constant of 1000 kJ/mol/nm. The obtained nanocrystal was subsequently solvated and further equilibrated for 90 ns in the *NpT* ensemble. A Bussi thermostat was adopted to maintain the temperature at 300 K.^45^ The pressure was maintained at 1 atm, using the Berendsen barostat followed by the Parrinello–Rahman approach.^46,47^ After this equilibration, several production runs of 50 ns each were performed until the potential energy became stable.

### Replica Exchange with Solute Tempering

For a single molecule, the replica exchange with solute tempering 2 (REST2) method was employed to enhance conformational sampling.^35^ Using classical MD simulations, a single polypeptoid molecule with explicit solvent molecules was first equilibrated for 5 ns to reach an equilibrated density. REST2 simulations were carried out as the next step in the *NVT* ensemble using a Bussi thermostat to maintain the temperature at 300 K.^45^ The temperature ladder was built by scaling the nonbonded and dihedral parameters of the polypeptoid using PLUMED (version 2.7.2).^48^ The temperatures covered the range from 300 to 600 K, and the exchange rates were ensured to be >25% for all replicas. A trajectory of 60 ns was collected for the aqueous systems. For the system solvated with tetrahydrofuran, the time step was reduced to 1 fs, and a trajectory of 40 ns was collected.

## Supporting Information Available

Starting structure for MD simulations; urea/water molar ratio near peptoid surface and SASA; RMSD for an isolated molecule in pure water, conformations of isolated H_2_-Ndc_10_-Nte_10_-NH_2_ and Ac-Ndc_10_-Nte_10_-NH_2_ in pure water; topologies of protonated N-terminus and trifluoroacetate; atom types and partial charges; and radial distribution functions for N-terminus and trifluoroacetate (PDF)
Protonated-Ndc10-Nte10 nanofiber in 4 M urea (PDB)
Protonated-Ndc10-Nte10 nanofiber in pure water (PDB)
Ac-Ndc10-Nte10-NH2 nanosheet in pure water (PDB)

## Supplementary Material

nn3c12228_si_001.pdf

nn3c12228_si_002.pdb

nn3c12228_si_003.pdb

nn3c12228_si_004.pdb
