# Supplementary material for: Thermodynamic Driving Forces for the Self-Assembly of Diblock Polypeptoids
Source: ACS Nano. 2024 May 29;18(23):14917–24. doi: 10.1021/acsnano.3c12228 (PMC11171762; doi:10.1021/acsnano.3c12228)
Supplement: Supplementary file 1 — nn3c12228_si_001.pdf [file nn3c12228_si_001.pdf]

Thermodynamic driving forces for the self-assembly of diblock polypeptoids

Xubo Luo[1], Tianyi Yu[1], Nan K. Li[1], Ronald N. Zuckermann[1,2], Xi Jiang[1], Nitash P. Balsara[1], David Prendergast[1,2]

[1] Materials Sciences Division, Lawrence Berkeley National Laboratory, Berkeley, CA, 94720 USA

[2] The Molecular Foundry, Lawrence Berkeley National Laboratory, Berkeley, CA 94720, USA

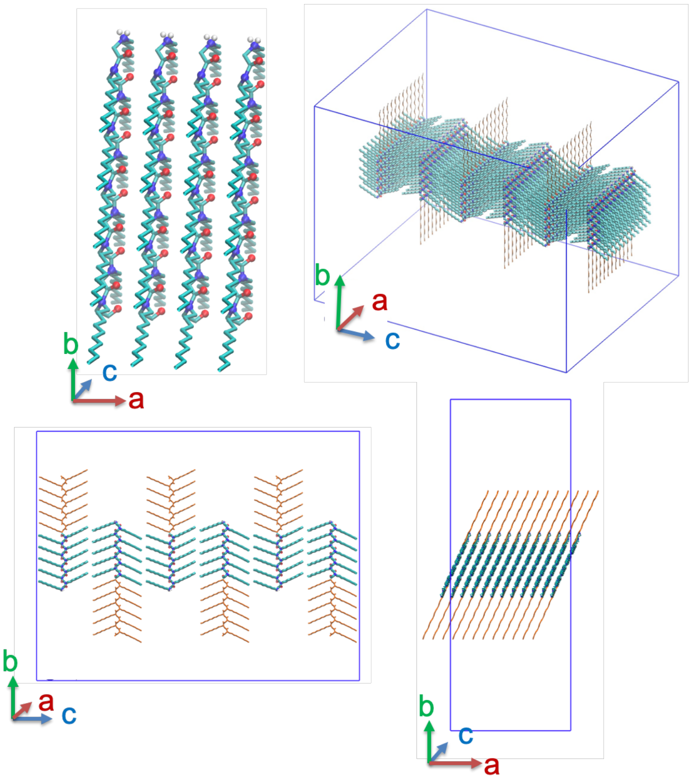

Figure S1. Starting structure for MD simulations. The molecular stacks adopt the all-cis conformation and the nanofiber with periodic boundaries in *a* direction has the tilt angle based on our preliminary tests.

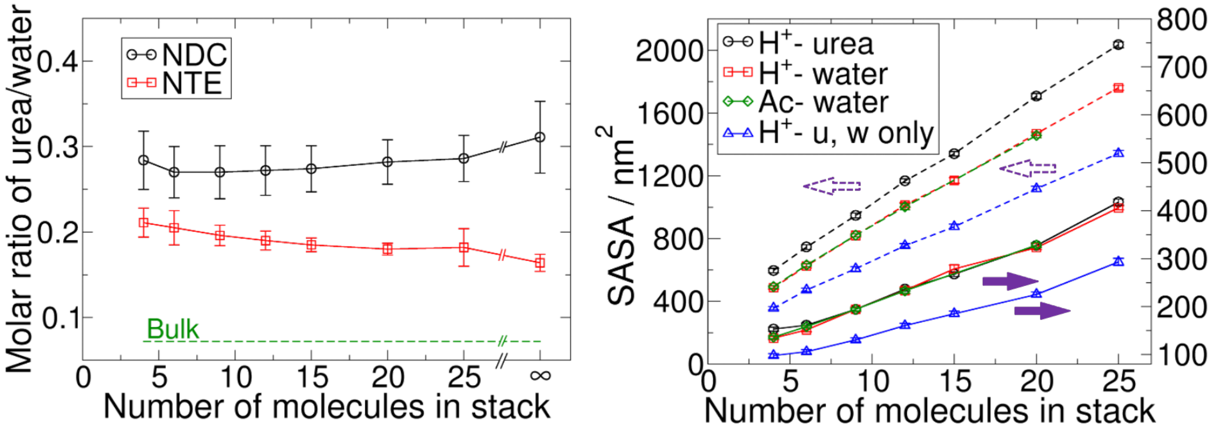

Figure S2. Urea/water molar ratio near peptoid surface and solvent accessible surface area (SASA). The probe of SASA measurement was set to 1.4 Å. Solid: Ndc only; Dash: Ndc and Nte; Blue line: Accessible to water, i.e., excluding the surface covered by urea within 3 Å.

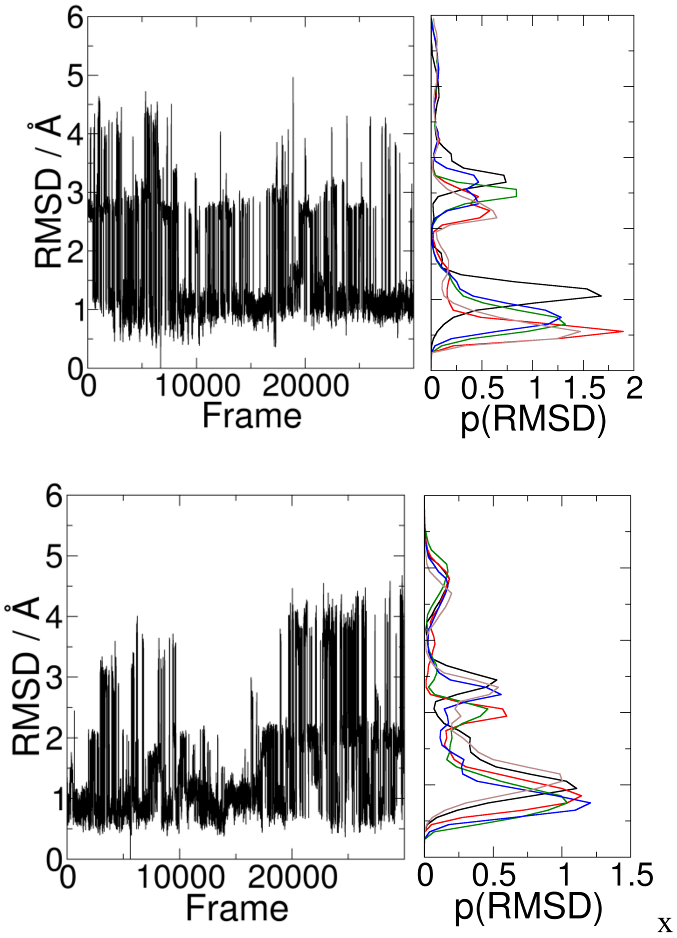

Figure S3. RMSD of all non-hydrogen backbone atoms an isolated H_2-Ndc_{10}-Nte_{10}-NH_2 (top) and Ac-Ndc_{10}-Nte_{10}-NH_2 (bottom) molecule in pure water. The distribution of RMSD was calculated with five different reference frames of coiled structure.

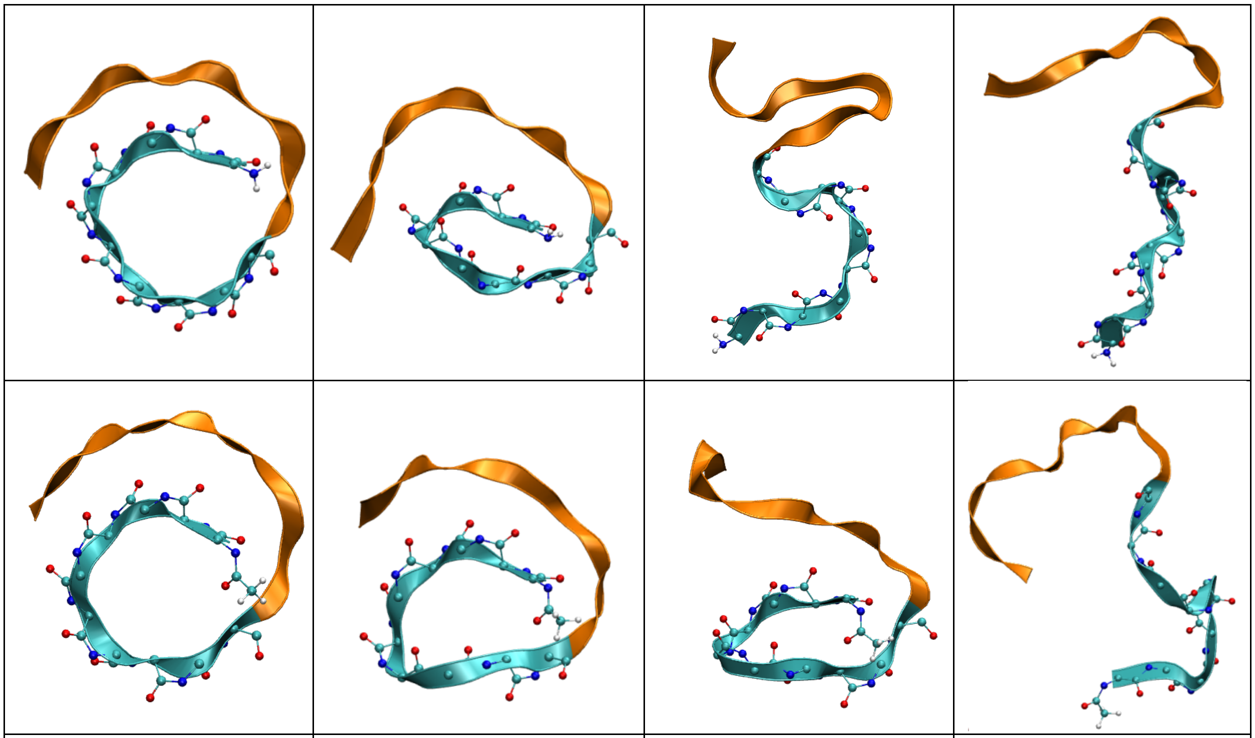

(A)	(B)	(C)	(D)

Figure S4. Conformations of isolated H_2-Ndc_{10}-Nte_{10}-NH_2 (upper) and Ac-Ndc_{10}-Nte_{10}-NH_2 (lower) in pure water. (A) coil; (B) and (C) semi-open; (D) open.

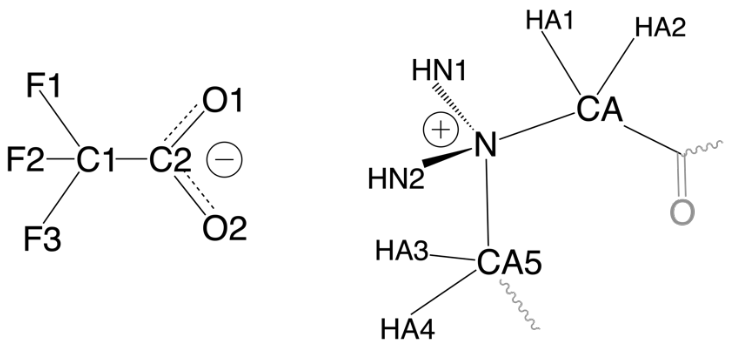

Figure S5. Topologies of protonated N-terminus and trifluoroacetate.

Table S1. Atom types and partial charges of protonated N-terminus and trifluoroacetate computed using B3LYP/6-311G**.

H^+-Nterm	Type	Charge / |e|
HN1/HN2	HGP1	0.26
N	NTOID	-0.07
CA	CG321	0.029
HA1/HA2	HGA2	0.123
CA5	CG321	0.029
HA3/HA4	HGA2	0.123
TFA^-	Type	Charge / |e|
F1/F2/F3	FGA3	-0.24
C1	CG302	0.46
C2	CG2O3	0.64
O1/O2	CG2D2	-0.69

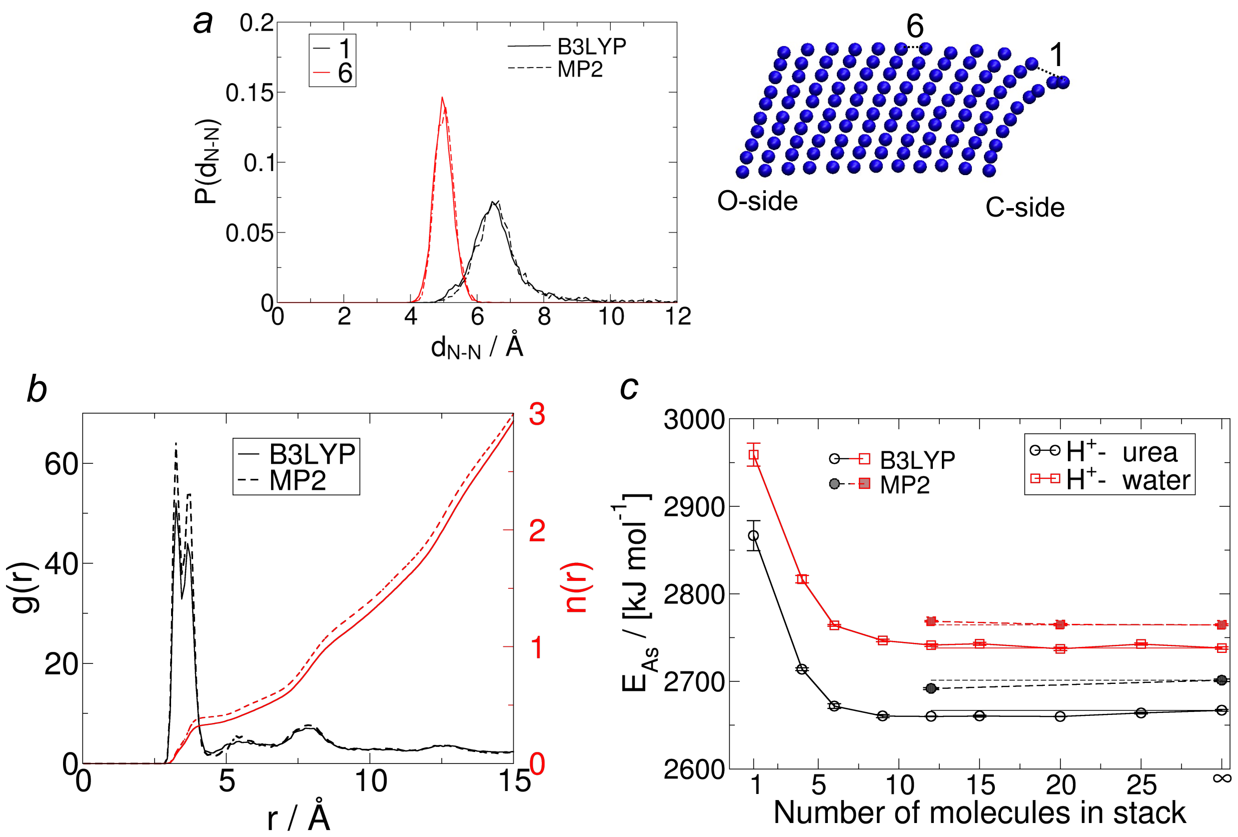

Figure S6. Test of an alternative set of partial charges for the protonated N-terminus and trifluoroacetate (TFA) calculated at the MP2/6-31G* level (**Table S2**) using Q-Chem 5.4.1.[1] **a.** N-termini separation at the C-side and the middle of a nanofiber, showing the peel-off at C-side. **b.** Radial distribution function (rdf) and coordination number for the nitrogen from the N-terminus and carbon from the trifluoromethyl group. **c.** Updated assembly energies using MP2 partial charges for nanofiber and nanosheet overlaid on the data for protonated peptoid assemblies from **Figure 1c** using B3LYP charges for TFA and protonated N-termini. The data of pure water was shifted by +40 kJ/mol for clarity. The N-termini separation and N-terminus-TFA rdf show very small differences with respect to that using B3LYP partial charges in the main text. The qualitative comparison of nanofiber and nanosheet has negligible change in terms of assembly energy. We conclude that the slightly altered partial charges obtained from MP2/6-31G* have a negligible impact, provided that the total charge of N-terminus and TFA are maintained at +1e and -1e.

Table S2. Alternative partial charges using MP2/6-31G*.

H^+-Nterm	Charge / |e|
HN1/HN2	0.272
N	0.06
CA	-0.087
HA1/HA2	0.156
CA5	-0.087
HA3/HA4	0.156
TFA^-	Charge / |e|
F1/F2/F3	-0.26
C1	0.54
C2	0.74
O1/O2	-0.75

Reference

(1) Shao, Y.; Gan, Z.; Epifanovsky, E.; et al. Advances in Molecular Quantum Chemistry Contained in the Q-Chem 4 Program Package. *Mol Phys* **2015**, *113*, 184–215.
